# Determination of *CD177* (human neutrophil antigen 2) polymorphisms using nanopore sequencing

**DOI:** 10.1111/vox.70020

**Published:** 2025-03-25

**Authors:** Kirstine Kløve‐Mogensen, Thure Mors Haunstrup, Anne‐Louise Fjordside Bilde, Rudi Steffensen

**Affiliations:** ^1^ Department of Clinical Immunology Aalborg University Hospital Aalborg Denmark; ^2^ Department of Clinical Medicine Aalborg University Aalborg Denmark; ^3^ Department of Clinical Medicine Aarhus University Aarhus Denmark

**Keywords:** CD177, genotyping, HNA‐2, nanopore sequencing

## Abstract

**Background and Objectives:**

Human neutrophil antigen 2 (HNA‐2), encoded by the *CD177* gene, is considered one of the most important neutrophil antigens in human medicine, but molecular testing of *CD177* is complicated and therefore not a standard procedure for investigating CD177 expression. CD177 expression can vary from 0% to 100%, and to date, the molecular basis for altered or non‐expressed genes has not been determined. Reliance on phenotyping and crossmatching to investigate these neutropenic clinical cases is inconvenient for patients and demands substantial resources within the laboratory. The purpose of this study was therefore to test a new molecular testing approach based on long‐read nanopore sequencing.

**Materials and Methods:**

DNA from 44 Danish blood donors with different levels of CD177 expression, 22 of whom were found to be CD177 null, was selected as test samples. All the DNA was sequenced for the first eight exons and the beginning of exon 9 of *CD177*.

**Results:**

All incidences of CD177 null cases could be associated with the known variant c.787A>T;p.K263X (rs20182172), and a correlation was observed between c.787A>T heterozygosity and a reduced expression of CD177, which is consistent with previously published findings. The c.1291G>A;p.G431R (rs78718189) variant was found to be linked to the atypical expression of CD177. The nanopore assay revealed a total of 14 variants in 7 exons in the 44 tested samples.

**Conclusion:**

On the basis of these observations, we conclude that long‐read nanopore sequencing can be a reliable tool for the routine laboratory molecular testing of *CD177*.

**Highlights**

- Long‐read nanopore sequencing is a reliable tool for *CD177* genotyping.
- All incidences of CD177 null cases were associated with the known variant c.787A>T.
- The c.1291G>A variant was linked to atypical expression of CD177.

## INTRODUCTION

Human neutrophil antigen 2 (HNA‐2) is encoded by the *CD177* gene. *CD177*, located on chromosome 19, contains nine exons and two untranslated regions and encodes a 437‐amino‐acid protein. CD177 is a glycosylphosphatidylinositol (GPI)‐anchored protein [1, 2]. HNA‐2 antibodies are involved in numerous disorders, including autoimmune neutropenia (AIN), neonatal alloimmune neutropenia (NAIN) and haematopoietic stem cell graft failure [3, 4, 5, 6, 7, 8]. Additionally, HNA‐2 is considered one of the most important neutrophil antigens in human medicine because of its involvement in transfusion‐related acute lung injury (TRALI) and other transfusion‐related pulmonary reactions [9, 10, 11, 12]. HNA‐2 typically has a bimodal expression pattern with both CD177‐positive and ‐null populations. The percentages of HNA‐2‐positive neutrophils range from 0% to 100%. Approximately 3%–5% of European, Brazilian and North American populations do not express HNA‐2, while this frequency is reported to be >10% in French and Western Japanese populations [13, 14]. Individuals who have HNA‐2 non‐expression, also referred to as HNA‐2 null individuals, are at risk of producing HNA‐2 isoantibodies when the HNA‐2 antigen is introduced through transfusion, pregnancy or bone marrow transplantation. The primary genetic mechanism of HNA‐2 null and HNA‐2 expression variation is caused by the nonsense single‐nucleotide polymorphism (SNP) c.787A>T;p.K263X (rs201821720), which is located in exon 7 [15]. Another SNP, c.1291G>A;p.G431R (rs78718189) in exon 9 of *CD177*, has been associated with the absence of the CD177 protein in c.787A>T heterozygous individuals, as well as with atypical expression of the HNA‐2 antigen on the neutrophil surface (three peaks: one negative peak and two positive peaks). The CD177 protein contains a short stretch of hydrophobic amino acids that forms the GPI signal. The polymorphism c.1291G>A is located within the carboxy‐terminal hydrophobic region of CD177. The amino acid change from glycine to arginine may affect the hydrophobicity of the CD177 GPI signal, leading to the destabilization of the CD177 protein from the neutrophil surface [16]. The c.787A‐c.1291A haplotype could lead to reduced HNA‐2 expression levels and/or the absence of HNA‐2 expression in human subjects [16]. Copy number variation (CNV) is rare for *CD177*, and three copies have been found in approximately 5%, whereas 0% have been found to have zero copies of the gene, even though you would expect there to be equilibrium between copy numbers [15]. Factors other than genetic variations in the coding regions of the *CD177* gene have been associated with the expression of CD177, including DNA methylation [17] and polymorphisms in the promoter region of transcription factor binding sites [18]. A recent study revealed an association of the CD177 null cell fraction with CpG methylation in the regulatory promoter region [19], but another confirmed the presence of CD177 messenger RNA (mRNA) in both CD177‐positive and CD177 null neutrophils, indicating the presence of an active gene in both subpopulations. Sequencing of CD177 mRNA revealed identical sequences in both subpopulations, rejecting gene silencing as the mechanism [14].

**FIGURE 1:**
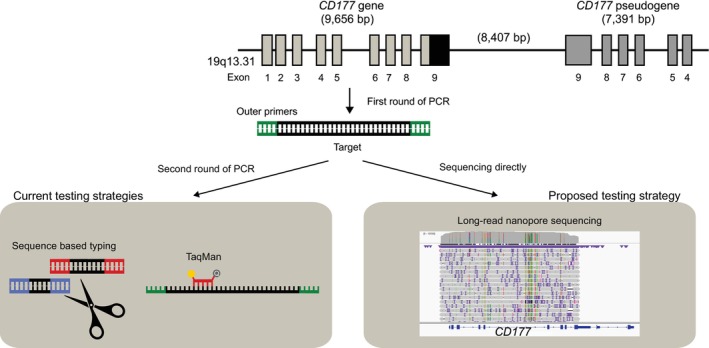
The structure and proximity of the *CD177* and *CD177P1* genes, and an illustration of the current amplification and testing strategies to obtain specificity for *CD177*, as well as the one proposed in this study.

Most laboratories determine CD177 expression with flow cytometry only, and a survey of HNA investigations published by Bayat et al. in 2023 revealed that only 5 out of 17 laboratories performed molecular testing of HNA‐2 [20]. Molecular approaches are complicated by a pseudogene (*CD177P1*), which is highly homologous to exons 4–9 of *CD177* but located on the opposite (minus) strand (Figure 1) [21, 22, 23]. Because of this, the most common way to determine HNA‐2 genotypes is to use long‐read polymerase chain reaction (PCR) to produce a long template for further investigation with either PCR‐sequence‐specific primers (PCR‐SSP) or PCR‐sequence‐based typing (PCR‐SBT) [15, 22, 23, 24, 25]. However, two rounds of PCR increase the risk of contamination during transferring first‐round amplification products to a second tube and demand more hands‐on time.

## MATERIALS AND METHODS

### Study cohort

**FIGURE 2:**
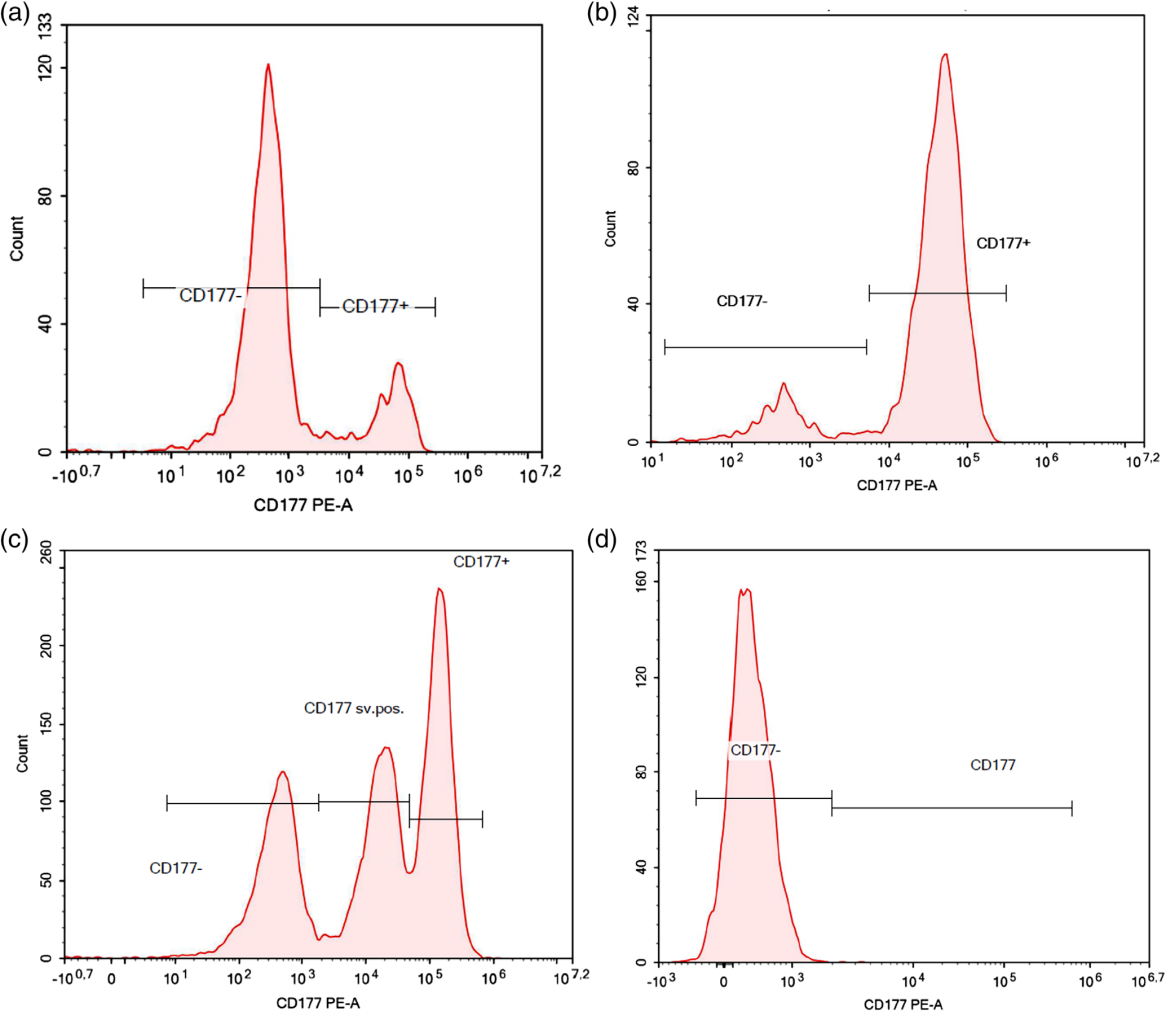
Examples of flow cytometry graphs when testing for CD177 with MEM‐166 staining. (a) 18% positive. (b) 85% positive. (c) Atypical expression. (d) Null.

A total of 44 samples representing as diverse a collection of phenotypes as possible were selected for sequencing. The 44 samples consisted of 22 CD177‐positive (AAL_001 to AAL_022) and 22 CD177‐null (AAL_023 to AAL_044) samples. The positive samples were selected because they presented the highest range of neutrophil expression, ranging from 18% to 85% positive cells (examples of flow‐GIFT patterns in Figure 2a,b). Five of the 22 positive samples (AAL_040 to AAL_044) presented an atypical expression pattern with two positive peaks (Figure 2c). Using the same criteria as those used in the literature [26], donors for whom fewer than 5% of granulocytes were positive according to flow cytometry analysis were called CD177‐null (Figure 2d). The Department of Clinical Immunology is the national centre for diagnostic neutrophil testing in Denmark and was the centre for sample collection in this study. All the samples were collected from healthy Danish blood donors, and the 22 CD177‐null samples were collected by screening >500 blood donors at different times. Written and oral informed consent from the participants were obtained according to the Danish Health Care Act and in accordance with the Declaration of Helsinki.

### HNA‐2 phenotyping

The samples used to test the long‐read nanopore sequencing assay were selected on the basis of their HNA‐2 phenotyping. The expression of HNA‐2 and the percentage of HNA‐2‐positive neutrophils were determined with an in‐house flow granulocyte immunofluorescence test (Flow‐GIFT) using phycoerythrin (PE) mouse anti‐human CD177, MEM‐166 (BD Biosciences). Leukocytes stained with either the PE‐conjugated anti‐CD177 monoclonal antibody (MEM‐166, mouse IgG1 [mIgG1]) (BD Biosciences, NJ, USA) or the mIgG1‐PE isotype control were analysed on a FACS Canto flow cytometer (BD Biosciences, NJ, USA) or NovoCyte 3000 flow cytometer (ACEA, San Diego, USA). FACSDiva software version 6.1.3 (BD Biosciences, NJ, USA) or NovoExpress software version 1.3.0, 1.4.1 or 1.5.4 (ACEA, San Diego, USA) was used to evaluate the flow cytometry data. Characteristic light‐scattering properties were used to identify neutrophils via flow cytometry.

### Long‐read nanopore sequencing

DNA was extracted from EDTA‐stabilized whole blood via the Maxwell RSC blood DNA kit on the Maxwell RSC instrument (Promega, Madison, WI, USA). The forward primers for *CD177*‐specific amplification, shown in Table 1, were designed with CLC Main Workbench 21 (Qiagen, Hilden, DE), and Ensembl [27] was used to confirm that the primer did not target the pseudogene *CD177P1*. The reverse primer was previously used by Wu et al. [16]. The amplicon (9248 bp) included the first eight exons and the beginning of exon 9. Each 20 μL PCR consisted of 200 ng of template DNA, 10 μL of long‐range PCR: GoTaq® Long PCR Master Mix (Promega, Madison, WI, USA) and 1 μL of primer mixture (final conc. 0.5 mM of each). PCR was performed on a Proflex™ (Applied Biosystems, Carlsbad, CA, USA) with the following conditions: 95°C for 2 min, 35 cycles of 94°C for 30 s, 63°C for 30 s, 65°C for 9 min and 1 cycle of 72°C for 10 min. PCR products were visualized via gel electrophoresis (1% agarose in Tris Borate EDTA buffer) and quantified with a Qubit FlexFluorometer (Thermo Fisher Scientific, Waltham, MA, USA) via a broad range kit (Thermo Fisher Scientific, Waltham, MA, USA). Barcoding and sequencing were performed with the rapid sequencing gDNA‐barcoding kit (SQK‐RBK110.96) using the protocol (version RBK_9126_v110_revO_24Mar2021) (Oxford Nanopore, Oxford, UK). Two hundred nanograms of amplification product were diluted to a volume of 7.5 μL, and 2.5 μL of barcoding mixture was added according to the manufacturer's protocol (Oxford Nanopore, Oxford, UK). The sample was cleaned with AMPure XP (Beckman Coulter, Brea, CA, USA) beads before pooling. Sequencing was performed with an R9.4 flow cell on a MinION Mk 1B sequencer (Oxford Nanopore, Oxford, UK). Basecalling was performed with guppy alignment software version 6.1.5 within the MinKNOW software during acquisition (Oxford Nanopore, Oxford, UK). Sequencing files were aligned to the human genome reference (Hg38) via minimap2 version 2.22 [28, 29], indexing was performed with the SAMtools index and coverage was determined with SAMtools coverage [30]. Manual analysis was performed via Integrated Genomics Viewer (IGV) version 2.16.1 [31, 32, 33, 34].

**TABLE 1:** Primer design for long‐read amplification of *CD177* (9248 bp).

Orientation	Sequence	Positioned	Genomic location	Melting temperature	Length	Source
Forward	GGGGGAACCTCGGGTCAAGATG	Before exon 1	19:43,353,123–43,353,144	63.6	22 bp	Designed with CLC
Revers	AGGTTGAGTGTGGGTGGTCAGC	Inside exon 9	19:43,362,350–43,362,371	64	21 bp	[16]

### Statistics

Statistical analysis was conducted via the statistical programme Stata (version 18.0, StataCorp, College Station, TX). SNP frequencies were estimated via direct counting. The expression patterns were compared via a chi‐square test, and the distributions were visualized via a boxplot.

## RESULTS

### Amplification and sequencing

**FIGURE 3:**
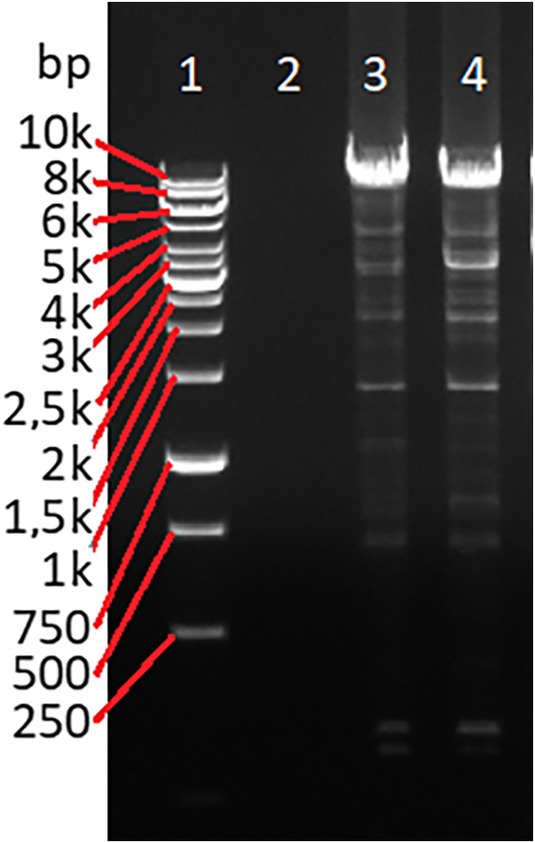
Gel electrophoresis (1% agarose) testing long‐range polymerase chain reaction amplification products of *CD177* (9248 bp). Lane 1: 10‐kb ladder; lane 2: no template control; lane 3: CD177 positive; lane 4: CD177 null.

Amplicons, with expected size 9248 bp, were confirmed for all 44 test samples by gel electrophoresis. Figure 3 shows a gel electrophoresis with a blank well containing the no template control (NTC) and two wells with bands between 8 and 10 kb containing amplicons from a sample that was CD177 positive and CD177 null for HNA‐2 expression. The sequence length of the shortest contig at 50% of the total assembly length, the N50, was 3.2 kb. The sequencing and mapping results for each sample are shown in Table 2. The number of reads aligned (after filtering) to the region (NC_000019.10: 43353145–43362349) varied from 959 to 32,325. The number of covered bases with depth ≥1 covered the whole region (9205 bases), resulting in a coverage of 100%. The mean depth coverage ranged from 124× to 12,117×. All samples produced high mean base qualities (21.2–25.1) and high mapping qualities (55.5–58.2). The sequencing results confirmed that the amplicons indeed aligned to the *CD177* gene.

**TABLE 2:** Nanopore sequencing results of *CD177* amplicons in the 44 tested samples.

ID	Number of reads	Covered bases	Coverage (%)	Mean depth of coverage	Mean baseQ	Mean mapQ
AAL_001	9396	9205	100	3869.7	21.2	57.8
AAL_002	24,482	9205	100	8488.0	22.2	55.7
AAL_003	24,394	9205	100	9595.4	22.0	57.7
AAL_004	23,599	9205	100	9052.9	22.1	57.2
AAL_005	16,638	9205	100	6099.0	22.1	56.8
AAL_006	20,142	9205	100	7551.7	22.0	57.0
AAL_007	22,234	9205	100	8647.3	22.0	57.4
AAL_008	16,837	9205	100	6481.7	22.1	57.3
AAL_009	15,904	9205	100	6308.2	22.1	57.6
AAL_010	28,687	9205	100	11,359.4	22.0	57.5
AAL_011	17,733	9205	100	7007.3	22.0	57.6
AAL_012	23,436	9205	100	8977.5	22.0	57.3
AAL_013	22,075	9205	100	8485.7	22.1	57.4
AAL_014	14,652	9205	100	5441.8	22.1	57.1
AAL_015	959	9205	100	124.5	25.1	56.4
AAL_016	12,778	9205	100	5331.4	22.1	58.1
AAL_017	12,300	9205	100	4816.6	22.1	57.6
AAL_018	17,996	9205	100	6711.0	22.0	57.0
AAL_019	13,268	9205	100	4773.3	22.1	56.6
AAL_020	18,266	9205	100	6898.5	22.1	57.0
AAL_021	19,634	9205	100	7119.7	22.1	56.9
AAL_022	28,553	9205	100	9765.9	21.7	55.5
AAL_023	15,437	9205	100	5277.1	21.8	55.7
AAL_024	32,325	9205	100	12,116.8	21.6	56.6
AAL_025	8396	9205	100	3088.3	21.8	56.7
AAL_026	4636	9205	100	1863.8	21.3	57.4
AAL_027	20,344	9205	100	7815.8	21.8	57.3
AAL_028	5813	9205	100	2208.7	21.8	57.5
AAL_029	8871	9205	100	3263.2	21.8	56.4
AAL_030	9389	9205	100	3878.1	21.5	58.2
AAL_031	2485	9205	100	919.9	21.9	56.8
AAL_032	21,467	9205	100	7669.9	21.7	56.2
AAL_033	12,652	9205	100	4889.0	21.6	57.2
AAL_034	19,684	9205	100	6996.1	21.8	56.4
AAL_035	13,979	9205	100	4622.2	21.7	55.4
AAL_036	9218	9205	100	3519.4	21.2	57.1
AAL_037	22,106	9205	100	7934.2	21.8	56.2
AAL_038	5233	9205	100	2073.6	21.3	57.5
AAL_039	4855	9205	100	1942.7	21.2	57.4
AAL_040	16,552	9205	100	5708.3	21.7	56.2
AAL_041	23,091	9205	100	7703.8	21.8	55.6
AAL_042	19,641	9205	100	7708.9	21.7	57.4
AAL_043	4841	9205	100	1929.4	21.3	57.5
AAL_044	12,689	9205	100	5033.5	22.1	57.3

### Genetic variations in the *CD177* gene

**FIGURE 4:**
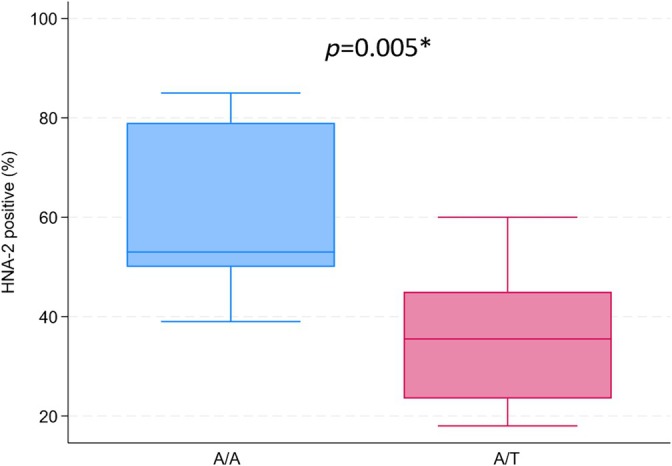
Comparison of the effect of c.787A>T on CD177 expression pattern in 17 samples. Blue: c.787A/A, *n* = 9 (AAL_031 to AAL_039) (min = 39; median = 35.5; max = 85). Red: c.787A/T, *n* = 8 (AAL_023 to AAL_030) (min = 18; median = 53; max = 60). *Significant statistical difference.

A total of 14 SNPs across seven exons of *CD177* were identified in the 44 test samples, as presented in Table 3. The presence of SNPs in the 22 CD177 null samples and the 22 CD177 positive samples is visualized in Tables 4 and 5. Twenty‐two samples were homozygous for the c.787A>T SNP, leading to a stop codon, corresponding to the 22 samples found to be CD177 null (AAL_023 to AAL_044). The c.787A>T SNP was in complete linkage disequilibrium (LD) with c.782G>A, c.786A>C, c.790G>A and c.799A>C, but LD was not observed among the other SNPs. Eight of the 17 (AAL_023 to AAL_030) samples positive and with a normal expression pattern for HNA‐2 were heterozygous for c.787A>T, and the remaining nine (AAL_031 to AAL_039) did not have the polymorphism. The influence of c.787A>T on expression patterns in positive samples is illustrated in the boxplot in Figure 4. The boxplot shows that c.787A>T heterozygosity affects gene expression, and the two groups were significantly different (*p* = 0.005). In the nine samples with c.787A/A, the percentage of positive neutrophils ranged from 39% to 85%, whereas in the samples with c.787A/T, the percentage ranged from 18% to 60%. All five positive samples with atypical expression (AAL_040 to AAL_044) were found to be heterozygous for the c.1291G>A polymorphism. This polymorphism was not detected in any of the other samples.

**TABLE 3:** Human neutrophil antigen 2 mutations found with nanopore sequencing in all 44 tested samples.

Region	Position (Hg38) Chr19	Rs no.	Variant	Amino acid change	Gene consequence	Prevalence, *n* = 44 (%)		
						Wildtype	Heterozygote	Homozygote
Exon 1	43,353,721	rs45441892	c.7G>C	p.A3P	Missense	16 (36.4)	18 (40.9)	10 (22.7)
Exon 2	43,353,892	rs45553433	c.92A>T	p.H31L	Missense	43 (97.7)	1 (2.3)	0 (0.0)
	43,353,914	rs45571738	c.114G>A	p.L38L	Synonymous	31 (70.5)	12 (27.3)	1 (2.3)
Exon 5	43,356,040	rs12981714	c.551T>G	p.V184G	Missense	39 (88.6)	4 (9.1)	1 (2.3)
	43,356,099	rs12980412	c.610G>A	p.D204N	Missense	34 (77.3)	5 (11.4)	0 (0.0)
	43,356,103	rs12981771	c.614T>G	p.M205R	Missense	38 (86.4)	5 (11.4)	1 (2.3)
Exon 6	43,360,396	rs10425835	c.751C>A	p.L251I	Missense	31 (70.5)	11 (25.0)	2 (4.5)
Exon 7	43,361,164	rs200660811	c.782G>A	p.G261A	Missense	14 (31.8)	8 (18.2)	22 (50.0)
	43,361,168	rs587670082	c.786A>C	p.T262T	Synonymous	14 (31.8)	8 (18.2)	22 (50.0)
	43,361,169	rs201821720	c.787A>T	p.K263X	Stop	14 (31.8)	8 (18.2)	22 (50.0)
	43,361,172	rs200145410	c.790G>A	p.G264S	Missense	14 (31.8)	8 (18.2)	22 (50.0)
	43,361,181	rs12978146	c.799A>C	p.T267A	Missense	14 (31.8)	8 (18.2)	22 (50.0)
Exon 8	43,361,540	rs17856829	c.1042G>A	p.A348T	Missense	14 (31.8)	8 (18.2)	22 (50.0)
Exon 9	43,362,297	rs78718189	c.1291G>A	p.G431R	Missense	39 (88.6)	5 (11.4)	0 (0.0)

**TABLE 4:** Genetic variants present in the 22 human neutrophil antigen 2 null samples with long‐read nanopore sequencing.

HNA‐2 null		Exon 1	Exon 2		Exon 5			Exon 6	Exon 7					Exon 8	Exon 9	
		c.7G>C	c.92A>T	c.114G>A	c.551G>T	c.610A>G	c.614G>T	c.751C>A	c.782G>A	c.786A>C	c.787A>T	c.790G>A	c.799A>C	c.1042G>A	c.1291G>A	
ID	HNA‐2 (%)	p.A3P	p.H31L	p.L38L	p.V184G	p.D204N	p.M205R	p.L251I	p.G261A	p.T262T	p.K263X	p.G264S	p.T267A	p.A348T	p.G431R
AAL_001	0	G/G	A/A	G/G	G/G	A/A	G/G	C/C	A/A	C/C	T/T	A/A	C/C	G/G	G/G
AAL_002	0	G/G	A/A	G/A	G/G	A/A	G/G	C/C	A/A	C/C	T/T	A/A	C/C	G/G	G/G
AAL_003	0	G/G	A/A	G/A	G/G	A/A	G/G	C/C	A/A	C/C	T/T	A/A	C/C	G/G	G/G
AAL_004	0	G/C	A/A	G/A	G/G	A/A	G/G	C/C	A/A	C/C	T/T	A/A	C/C	G/G	G/G
AAL_005	0	G/C	A/A	G/A	G/G	A/A	G/G	C/C	A/A	C/C	T/T	A/A	C/C	G/G	G/G
AAL_006	0	C/C	A/A	G/G	G/G	A/A	G/G	C/C	A/A	C/C	T/T	A/A	C/C	G/G	G/G
AAL_007	0	G/C	A/A	G/G	G/T	A/G	G/T	C/C	A/A	C/C	T/T	A/A	C/C	G/G	G/G
AAL_008	0	G/C	A/A	G/A	G/G	A/A	G/G	C/C	A/A	C/C	T/T	A/A	C/C	G/G	G/G
AAL_009	0	C/C	A/A	G/G	G/G	A/A	G/G	C/C	A/A	C/C	T/T	A/A	C/C	G/G	G/G
AAL_010	0	G/C	A/A	G/G	G/G	A/A	G/G	C/C	A/A	C/C	T/T	A/A	C/C	G/G	G/G
AAL_011	0	G/G	A/A	G/G	T/T	G/G	T/T	C/C	A/A	C/C	T/T	A/A	C/C	G/G	G/G
AAL_012	0	G/C	A/A	G/G	G/G	A/A	G/G	C/C	A/A	C/C	T/T	A/A	C/C	G/G	G/G
AAL_013	0	C/C	A/A	G/A	G/G	A/A	G/G	C/C	A/A	C/C	T/T	A/A	C/C	G/G	G/G
AAL_014	0	C/C	A/A	G/A	G/G	A/A	G/G	C/C	A/A	C/C	T/T	A/A	C/C	G/G	G/G
AAL_015	0	C/C	A/A	G/G	G/G	A/A	G/G	C/C	A/A	C/C	T/T	A/A	C/C	G/G	G/G
AAL_016	0	C/C	A/A	G/G	G/G	A/A	G/G	C/C	A/A	C/C	T/T	A/A	C/C	G/G	G/G
AAL_017	0	G/G	A/A	G/G	G/G	A/A	G/G	C/C	A/A	C/C	T/T	A/A	C/C	G/G	G/G
AAL_018	0	G/C	A/A	G/G	G/G	A/A	G/G	C/C	A/A	C/C	T/T	A/A	C/C	G/G	G/G
AAL_019	0	G/C	A/A	G/G	G/G	A/A	G/G	C/C	A/A	C/C	T/T	A/A	C/C	G/G	G/G
AAL_020	0	G/C	A/A	G/A	G/G	A/G	G/T	C/C	A/A	C/C	T/T	A/A	C/C	G/G	G/G
AAL_021	0	G/C	A/A	G/A	T/G	A/A	G/G	C/C	A/A	C/C	T/T	A/A	C/C	G/G	G/G
AAL_022	0	C/C	A/A	G/G	G/G	A/A	G/G	C/C	A/A	C/C	T/T	A/A	C/C	G/G	G/G

*Note*: Colours: wildtype = green; heterozygotes = yellow; homozygote = red.

Abbreviation: HNA‐2, human neutrophil antigen 2.

**TABLE 5:** Genetic variants present in the 22 human neutrophil antigen 2 positive samples with long‐read nanopore sequencing.

HNA‐2 positive		Exon 1	Exon 2		Exon 5			Exon 6	Exon 7					Exon 8	Exon 9	
		c.7G>C	c.92A>T	c.114G>A	c.551G>T	c.610A>G	c.614G>T	c.751C> A	c.782G>A	c.786A>C	c.787A>T	c.790G>A	c.799A>C	c.1042G>A	c.1291G>A	
ID	HNA‐2 (%)	p.A3P	p.H31L	p.L38L	p.V184G	p.D204N	p.M205R	p.L251I	p.G261A	p.T262T	p.K263X	p.G264S	p.T267A	p.A348T	p.G431R
AAL_023	18	G/G	A/A	G/A	G/G	A/A	G/G	C/A	G/A	A/C	A/T	G/A	A/C	G/G	G/G
AAL_024	22	G/C	A/A	G/G	G/G	G/A	G/T	C/C	G/A	A/C	A/T	G/A	A/C	G/G	G/G
AAL_025	25	G/G	A/A	G/G	G/G	A/A	G/G	C/C	G/A	A/C	A/T	G/A	A/C	G/A	G/G
AAL_026	31	G/G	A/A	G/G	G/G	A/A	G/G	C/C	G/A	A/C	A/T	G/A	A/C	G/A	G/G
AAL_027	40	G/C	A/A	G/G	G/G	A/A	G/G	C/A	G/A	A/C	A/T	G/A	A/C	G/G	G/G
AAL_028	44	G/C	A/A	G/G	G/T	G/A	G/T	C/A	G/A	A/C	A/T	G/A	A/C	G/G	G/G
AAL_029	46	C/C	A/A	G/G	G/T	G/A	G/T	C/A	G/A	A/C	A/T	G/A	A/C	G/G	G/G
AAL_030	60	C/C	A/A	G/G	G/G	A/A	G/G	C/C	G/A	A/C	A/T	G/A	A/C	G/A	G/G
AAL_031	39	G/G	A/A	A/A	G/G	A/A	G/G	C/C	G/G	A/A	A/A	G/G	A/A	A/A	G/G
AAL_032	42	G/G	A/A	G/G	G/G	A/A	G/G	C/A	G/G	A/A	A/A	G/G	A/A	G/A	G/G
AAL_033	50	G/G	A/A	G/G	G/G	A/A	G/G	C/A	G/G	A/A	A/A	G/G	A/A	G/A	G/G
AAL_034	50	G/G	A/A	G/G	G/G	A/A	G/G	A/A	G/G	A/A	A/A	G/G	A/A	G/G	G/G
AAL_035	53	C/C	A/A	G/G	G/G	A/A	G/G	C/C	G/G	A/A	A/A	G/G	A/A	A/A	G/G
AAL_036	75	G/G	A/A	G/G	G/G	A/A	G/G	C/A	G/G	A/A	A/A	G/G	A/A	G/A	G/G
AAL_037	79	C/C	A/A	G/G	G/G	A/A	G/G	C/C	G/G	A/A	A/A	G/G	A/A	G/A	G/G
AAL_038	84	G/G	A/A	G/G	G/G	A/A	G/G	A/A	G/G	A/A	A/A	G/G	A/A	G/G	G/G
AAL_039	85	G/C	A/A	G/G	G/G	A/A	G/G	C/A	G/G	A/A	A/A	G/G	A/A	G/G	G/G
AAL_040	27/65	G/C	A/A	G/A	G/G	A/A	G/G	A/A	G/G	A/A	A/A	G/G	A/A	G/G	G/A
AAL_041	28/35	G/C	A/A	G/G	G/G	A/A	G/G	C/A	G/G	A/A	A/A	G/G	A/A	G/A	G/A
AAL_042	30/42	G/C	A/A	G/G	G/G	A/A	G/G	C/A	G/G	A/A	A/A	G/G	A/A	G/A	G/A
AAL_043	39/14	G/G	A/A	G/G	G/G	A/A	G/G	C/A	G/G	A/A	A/A	G/G	A/A	G/A	G/A
AAL_044	40/30	G/G	A/T	G/A	G/G	A/A	G/G	A/A	G/G	A/A	A/A	G/G	A/A	G/G	G/A

*Note*: Colours: wildtype = green; heterozygote = yellow; homozygote = red.

Abbreviation: HNA‐2, human neutrophil antigen 2.

## DISCUSSION

The assay designed for long‐read sequencing was able to successfully produce amplification products in all 44 test samples. Despite an amplification time of ~6 h, this approach demands minimal hands‐on time for both amplification and preparation for sequencing. There is also a lower risk of contamination than when two rounds of PCR are performed, and the sequencing time can be as low as 1–2 h per sample, depending on the state of the flowcell and the desired coverage. The number of reads obtained in a sample ranged from 959 to 32,325, with a mean depth of coverage ranging from 125× to 12,117×. Even with a lower number of reads, the high mean baseQ was >21 (a quality score of 20 [Q20] represents an error rate of 1 in 100 [meaning every 100 bp sequencing read may contain an error], with a corresponding call accuracy of 99%) and the mapQ was >55 (mapping quality ranges from 0 to 60 and a score of 50 is equal to an expected error of 1 in 100,000, or a mapping accuracy of 99.999%). We did not establish a minimum acceptable read depth, but we can conclude that we were able to obtain high‐quality data among all tested samples.

This is to our knowledge the first study of this size in which all tested CD177 null samples had the same genetic explanation; homozygosity for c.787T in *CD177*, which results in a premature stop codon (p.Lys263Ter). This mutation has previously been shown either in combination with c.997delG (compound heterozygote) or alone (homozygote) to be responsible for the absence of CD177 in HNA‐2 null individuals [5, 14, 15]. None of the 44 samples tested in this study had the c.997delG mutation, and all the samples that were heterozygous for c.787A>T were CD177 positive. A British study with approximately the same sample size as this study tested 21 CD177 null samples and reported that only 16/21 (76%) were c.787T homozygotes [35]. Additionally, they reported two other SNPs responsible for stop codons, c.1021C>T;p.Arg341Ter (rs201040394) and c.1254G>A;p.Trp418Ter (rs188387562), but the causative mutation/polymorphism for the remaining 5/21 samples could not be elucidated from the exon and adjacent intronic sequences. Their results were in agreement with a larger multi‐centre study combining 54 HNA‐2 null individuals of European origin that identified c.787T homozygosity in 43/54 (80%) [5]. Further studies are needed to determine whether the uniformity of the causative SNP in the CD177 null individuals in this study is a result of testing techniques or a small study population. A larger study is also needed to elucidate the percentage of CD177 null individuals and the frequency of SNPs in *CD177* among the Danish population. The *CD177* c.787A>T substitution has not only been associated with CD177 null but has also been shown to affect the distribution of CD177‐positive and CD177 null neutrophils in individuals with a bimodal expression pattern. Consistent with previous findings [5, 15], even though our study was based on a low number of samples, we observed that heterozygosity for the mutation was statistically significantly associated with a lower number of CD177‐positive neutrophils. However, the wide ranges (39%–85% for c.787AA and 18%–60% for c.787AT) indicate that other factors could also be involved. Importantly, we selected samples for the study cohort with the highest possible ranges of CD177 expression, and the distribution is therefore not representative of the Danish population.

The c.1291G>A polymorphism in exon 9 of the *CD177* gene has previously been associated with the absence of CD177 in individuals who are also heterozygous for c.787A>T, but it has also been associated with atypical expression (three peaks: one negative peak and two positive peaks) of CD177 on the neutrophil surface [14]. Among our 22 CD177‐positive samples, 5 presented atypical expression, which could be explained by heterozygosity for c.1291G>A.

In addition to c.787A>T and c.1291G>A, we also found 12 other variants, all of which have also been observed among British blood donors in Browne et al. [35], but their influence on HNA‐2 expression is yet to be determined. However, since these 44 test samples do not represent a normal Danish population but instead have been selected with the sole purpose of testing this assay, the frequencies of the variants are not representative of the frequency in the Danish population. It does, however, show the ability of this assay to find novel variants and the possibility of seeking genetic variants in samples that differ from those described here. Besides being able to allocate all HNA‐2 null non‐expression cases to one single SNP, this study did not identify any new SNPs and repeated already reported results from previous studies using other techniques [15, 16, 25].

Overall, long‐read nanopore sequencing was shown to be a robust technique for *CD177* genotyping, and all phenotypical expression patterns found with Flow‐GIFT in the test samples could be explained by genetic variations. With respect to the risk of isoantibodies, the ability to determine CD177 null is of greatest clinical value, and even though all 22 CD177 null samples had the same genetic variation to explain their phenotypic behaviour, this assay has the possibility of identifying other described null genetic variants.

This method can enable a more rapid turnaround time in suspected cases and have the possibility of reducing or negating the need for fresh granulocytes for phenotyping and crossmatch studies. CD177 null identification by genotyping could lessen the inconvenience for patients, their families and clinicians to provide subsequent, time‐sensitive samples while permitting the identification of potential antigenic polymorphisms.

## CONFLICT OF INTEREST STATEMENT

The authors declare no conflicts of interest.

## Data Availability

The data that support the findings of this study are available from the corresponding author upon reasonable request.
